# Resolving the Trophic Relations of Cryptic Species: An Example Using Stable Isotope Analysis of Dolphin Teeth

**DOI:** 10.1371/journal.pone.0016457

**Published:** 2011-02-18

**Authors:** Kylie Owen, Kate Charlton-Robb, Ross Thompson

**Affiliations:** 1 Australian Centre for Biodiversity and School of Biological Sciences, Monash University, Victoria, Australia; 2 Centre for Environmental Stress and Adaptation Research (CESAR), Monash University, Victoria, Australia; Institut Pluridisciplinaire Hubert Curien, France

## Abstract

Understanding the foraging ecology and diet of animals can play a crucial role in conservation of a species. This is particularly true where species are cryptic and coexist in environments where observing feeding behaviour directly is difficult. Here we present the first information on the foraging ecology of a recently identified species of dolphin (Southern Australian bottlenose dolphin (SABD)) and comparisons to the common bottlenose dolphin (CBD) in Victoria, Australia, using stable isotope analysis of teeth. Stable isotope signatures differed significantly between SABD and CBD for both δ^13^C (−14.4‰ vs. −15.5‰ respectively) and δ^15^N (15.9‰ vs. 15.0‰ respectively), suggesting that the two species forage in different areas and consume different prey. This finding supports genetic and morphological data indicating that SABD are distinct from CBD. In Victoria, the SABD is divided into two distinct populations, one in the large drowned river system of Port Phillip Bay and the other in a series of coastal lakes and lagoons called the Gippsland Lakes. Within the SABD species, population differences were apparent. The Port Phillip Bay population displayed a significantly higher δ^15^N than the Gippsland Lakes population (17.0‰ vs. 15.5‰), suggesting that the Port Phillip Bay population may feed at a higher trophic level - a result which is supported by analysis of local food chains. Important future work is required to further understand the foraging ecology and diet of this newly described, endemic, and potentially endangered species of dolphin.

## Introduction

Understanding the foraging ecology of a species or population can strongly impact on the ability to accurately conserve the species or population of concern. This problem is particularly acute when species are difficult to differentiate in the field, and where feeding occurs in habitats where direct observation of diet is not possible. Cryptic species can arise where long term differences in diet generate habitat segregation and eventual reproductive isolation. Differences in foraging ecology can allow distinctions between species even when they are superficially similar [1]. A number of studies have now shown that underlying differences in diet can drive speciation in groups as diverse as birds [2], molluscs [3], fish [4] and mammals. For example, populations of killer whales in the northwest Pacific exhibit distinct differences in dietary preferences, with resident populations targeting salmon, while transient populations forage on other marine mammals and sea birds [5]. This distinction in foraging ecology has long been used as an argument for differences in the way that the two populations should be managed [6] as well as an additional line of evidence supporting divergence of two populations as separate species [7].

Bottlenose dolphins are a cosmopolitan species that has adapted to many different environments around the world. They are considered to be an opportunistic predator with a wide and varied diet [8]–[11]. In Australia, bottlenose dolphins have been divided into two species; the Indo-Pacific bottlenose dolphin (*Tursiops aduncus*) which occurs inshore (<1 km from the coast), and the common bottlenose dolphin (CBD) (*Tursiops truncatus*) which is rarely seen in coastal waters and is distributed offshore [12]. Until recently in south eastern Australia, the offshore animals have been described as CBD and the inshore animals were presumed to be Indo-Pacific bottlenose dolphins [13]. However recent genetic research has found strong evidence that inshore populations are in fact a new species of dolphin (the southern Australian bottlenose dolphin (SABD), which is likely to be endemic to the area [14]–[16]. The SABD appears to form distinct inshore populations with one being found in a large drowned river system of Port Phillip Bay and the other found in a series of coastal lakes and lagoons called Gippsland Lakes. Both of these population sizes are small with ongoing population studies suggesting that the southern Port Phillip Bay population is comprised of approximately 80 individuals and the Gippsland Lakes population is comprised of approximately 50 individuals (Dolphin Research Institute; Charlton-Robb, *et al.*, unpublished data). To date there have been no published studies on the diet and foraging ecology of the SABD or ecological differences between the SABD and CBD.

One of the challenges in assessing diet in many animals is that they are not often observed feeding and that analysis of faecal remains or stomach contents is often biased. This issue has been noted for a wide range of animals including birds [17], mammals [18] and crustaceans [19]. In marine mammals determining diet via direct observation has long presented researchers with difficulties, as feeding occurs underwater, and observations of feeding do not necessarily represent overall diet [20]. While gut content analysis can provide insight to the prey ingested [11], [21] prey items are assimilated at different rates and this can give a false indication of the diet of the animal [22]–[25]. Stranded dolphins often have empty stomachs, or have gut contents that may reflect the feeding of the animal while ill or stressed prior to stranding. The only information collected to date on diet for CBD and SABD in south eastern Australia has been through direct observations of feeding, the association of dolphin distribution patterns with known fisheries areas, and gut contents of a few stranded dolphins, all of which have limitations when assessing diet. Collectively, and in combination with the very low population size of SABD, these issues make it extremely difficult to determine differences in feeding ecology between SABD and CBD.

Stable isotope analysis compares differences in the ratios of isotopes of elements found in the tissues of prey and predators to gain information on diet. Ratios of the two stable isotopes of nitrogen (^14^N/^15^N, hereafter δ^15^N) provides information on trophic position, as ^14^N is excreted preferentially by organisms resulting in an accumulation of the heavier ^15^N isotope up the food chain [23], [26]. The amount of fractionation between trophic levels varies depending on the system being studied but is approximately 3‰ per trophic level [27], [28]. The trophic level of animals can therefore be estimated by comparing the δ^15^N value of consumers to that of potential prey. Ratios of stable carbon isotopes (^12^C/^13^C, hereafter δ^13^C) vary between different basal resources, and have only slight fractionation between trophic levels [29]. As a consequence, δ^13^C values indicate the source of the primary production in the food chain leading to the consumer [30]. Where food chains are based equally on two sources with differing δ^13^C signatures, consumer δ^13^C values will be intermediate between sources.

Recently there has been an increasing appreciation of the ability of stable isotopes to identify movements of animals between habitats and geographical locations [31]. There is variability in δ^13^C values in aquatic ecosystems between offshore, inshore, benthic, and pelagic habitats [20], [32]–[34]. Coastal environments are more enriched in ^13^C relative to offshore environments [35]. These differences allow the foraging zones of marine mammals to be determined [31], [35]. Therefore, isotopes may be used to determine differences between animals feeding in different areas and on different prey. This approach is likely to be particularly powerful where cryptic species coexist but rely on different resources. In addition, stable isotopes can be applied on a range of different tissues, including muscle, bone, hair and teeth, allowing their application to material which is sub-fossil, preserved or from contemporary specimens. This is particularly valuable in mammals, where there are often significant amounts of material in museum collections, and where habitat loss, degradation and fragmentation may not allow meaningful interpretation of current diets.

The use of stable isotope analysis has become more common across a range of mammalian groups. There are now published studies of putative trophic links from a wide range of species, including bovids [36], marsupials [37] and bears [38]. Although many different tissues may be used for stable isotope analysis, teeth have a slow turnover rate and are believed to represent an average of an entire lifetime's diet due to the way that they grow [26], [30], [39]. Additionally, organic material is preserved well in teeth and historic samples can be obtained from museums to analyse how diet and foraging location have changed over time [20]. This approach has been applied to a range of marine mammals in recent years. For example, it has been shown that an increase in trophic level and a change in foraging location occurred in populations of Steller sea lions in Alaska between 1960 and 1980 [40]. Similarly, the teeth of marine mammals can be used to study differences in diet between age classes, genders, geographical locations and time periods [20]. As dolphin teeth grow continuously throughout their lifetime, they are ideal for analysing changes in diet through the life of individuals. Dolphin calves are born with hollow teeth and as they grow, new layers are added to the interior of the tooth until the pulp cavity is filled. This allows identification of diet both as an integrated average through the life of the animal, but also through different life stages, through analysis of different parts of the teeth.

**Figure 1:**
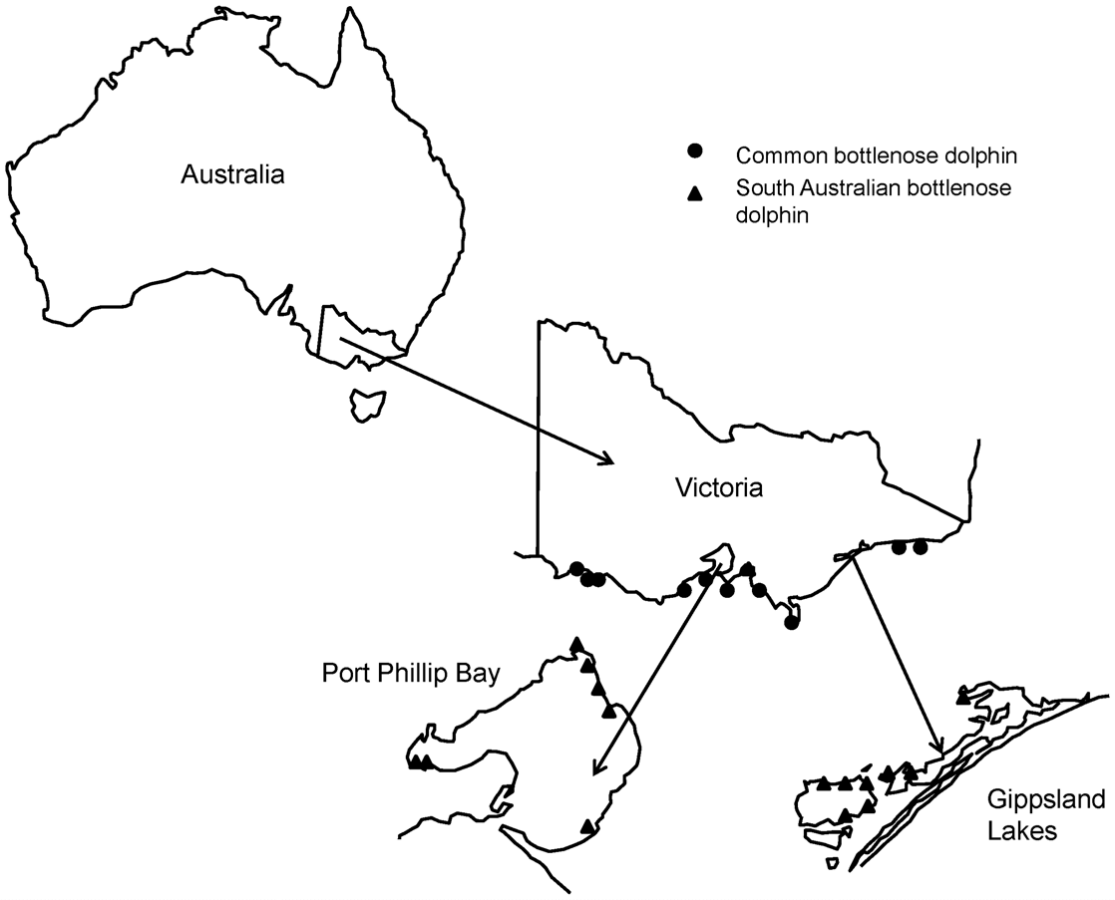
Location of tooth sample collections of common bottlenose dolphins (CBD) and south Australian bottlenose dolphins (SABD). It can be seen that the CBD strandings occur on beaches open to offshore waters, whereas the SABD mainly strands in either Port Phillip Bay or the Gippsland Lakes.

This study aimed to determine whether any differences in diet or foraging ecology occur between SABD and CBD in south-eastern Australia using stable isotope analysis of teeth. The SABD is commonly found in coastal areas of Port Phillip Bay and the Gippsland Lakes whereas the CBD is rarely seen in these coastal areas and more regularly observed in the offshore areas of Bass Strait. These observations of dolphin species sightings are also supported by the locations of strandings of both of these species (Figure 1) with the SABD commonly stranding in inshore coastal waters of Port Phillip Bay and the Gippsland Lakes, whereas the CBD regularly strands on beaches exposed to offshore waters. Therefore, it is hypothesised that SABD are more reliant on coastal prey sources than CBD which may rely more heavily on offshore food sources. Additionally, it is hypothesised that given the distinct environmental differences between the Gippsland Lakes and Port Phillip Bay, the dolphins in the two populations of SABD will have different diets. Within the SABD it has been observed that the active foraging strategy of the Gippsland Lakes population differs from that of the Port Phillip Bay population (Dolphin Research Institute; Charlton-Robb, *et al.*, unpublished data). Also, there is a distinct difference in the pattern of tooth wear between the two populations of SABD (Dolphin Research Institute; Charlton-Robb, *et al.*, unpublished data). These observed differences suggest that a difference in diet is also likely between the two populations of SABD.

## Results

**Figure 2:**
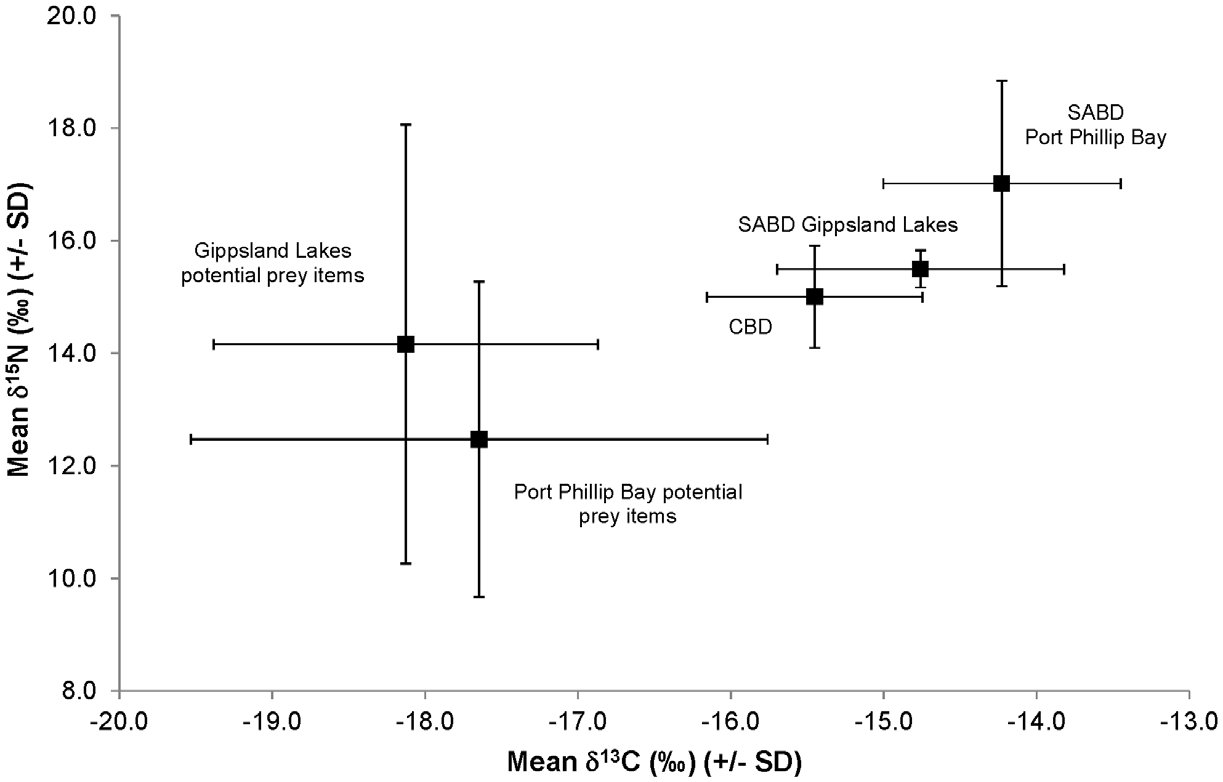
Species and population differences in stable isotope signatures and comparisons to potential prey items in Port Phillip Bay and the Gippsland Lakes. δ^13^C (‰) and δ^15^N (‰) signatures (mean +/- SD) for common bottlenose dolphins (CBD) (n = 17) and the southern Australian bottlenose dolphin (SABD) in Port Phillip Bay (n = 5) and the Gippsland Lakes (n = 6) in Victoria, Australia are shown. Additionally the δ^13^C (‰) and δ^15^N (‰) signatures (mean +/- SD) for potential prey items in the Gippsland Lakes and Port Phillip Bay are shown.

The stable isotope signatures of the potential prey items ranged between −14.2‰ and −20.7‰ for δ^13^C and 9.0‰ to 23.5‰ for δ^15^N (Table 1). The Port Phillip Bay potential prey items had higher average δ^13^C (−17.6‰ vs. −18.1‰) and a lower average δ^15^N (12.5‰ vs. 14.2‰) compared to the Gippsland Lakes potential prey items (Figure 2).

**Table 1:** Stable isotope results for the prey items collected from the Gippsland Lakes and Port Phillip Bay.

Location	Prey Species	Mean δ^13^C+/- SD(‰)	Mean δ^15^N+/- SD (‰)	n	C:N
Gippsland Lakes	Snapper (*Pagrus auratus*)	−17+/- 0.1	22.9+/- 0.9	2	3:7
	Yellowfin Bream (*Acanthopagrus australis*)	−17.6+/- 0.6	14.3+/- 0.2	2	3:6
	Squid (*Nototodarus gouldi*)	−19.7+/- 0.0	12.8+/- 0.3	2	3:8
	Silver Trevally (*Pseudocaranx dentex*)	−16.6+/- 0.5	10.6+/- 0.4	3	3:7
	Flathead (*Platycephalus sp.*)	−19+/- 0.1	13.9+/- 0.2	2	3:7
	Garfish (*Hyporhampus sp.*)	−19.2+/- 0.4	12.9+/- 0.7	3	3:6
Port Phillip Bay	Snapper (*Pagrus auratus*)	−17.4+/- 0.4	12.8+/- 0.1	2	3:7
	Yellowfin Bream (*Acanthopagrus australis*)	−19.3+/- 2.0	16.6+/- 6.2	2	3:7
	Squid (*Nototodarus gouldi*)	−19.1+/- 0.5	12.1+/- 0.3	3	3:7
	Silver Trevally (*Pseudocaranx dentex*)	−17.7+/- 2.1	12+/- 1.8	2	3:6
	Flathead (*Platycephalus sp.*)	−15.6+/- 1.1	11.1+/- 0.2	2	3:6
	Garfish (*Hyporhampus sp.*)	−16.2+/- 2.8	11+/- 2.2	2	3:6

C: N is represented as a mass ratio.

For both the CBD and the SABD no significant effect of age of the animal or year of sample collection was found in correlation to isotope values (Table 2 and Table 3). Therefore the results received are unlikely to have been influenced by the age of the animals or year of sample collection. Dolphin stable isotope signatures ranged between −12.7‰ to −15.9‰ for δ^13^C and 12.8‰ to 18.6‰ for δ^15^N. The CBD had significantly lower values for δ^13^C (−15.5‰ vs. −14.4‰) (F_1, 31_  = 14.515, p<0.001) and δ^15^N (15.0‰ vs. 15.9‰) (F_1, 31_  = 4.980, p = 0.033) compared to the SABD (Figure 2).

**Table 2:** Impact of age of the dolphins on stable isotope signatures.

Location	Isotope	r^2^	df	p	t
CBD	δ^13^C	0.071	13	0.337	0.996
	δ^15^N	0.007	13	0.767	−0.303
SABD (Gippsland Lakes)	δ^13^C	0.035	6	0.657	0.467
	δ^15^N	<0.001	6	0.981	−0.024
SABD (Port Phillip Bay)	δ^13^C	0.461	3	0.207	−1.603
	δ^15^N	0.320	3	0.320	1.189

Age variation observed in δ^13^C and δ^15^N for the populations of the south Australian bottlenose dolphins (SABD) in Port Phillip Bay the Gippsland Lakes and the common bottlenose dolphin (CBD), determined by linear regression analysis.

**Table 3:** Impact of year of specimen collection on stable isotope signatures.

Location	Isotope	r^2^	df	p	t
CBD	δ^13^C	0.006	16	0.767	0.301
	δ^15^N	0.014	16	0.637	0.481
SABD (Gippsland Lakes)	δ^13^C	0.190	6	0.281	−1.185
	δ^15^N	0.142	6	0.358	0.996
SABD (Port Phillip Bay)	δ^13^C	0.743	4	0.027*	−3.397
	δ^15^N	0.390	4	0.185	1.601

Temporal variation observed in δ^13^C and δ^15^N for the South Australian bottlenose dolphin (SABD) populations in Port Phillip Bay the Gippsland Lakes and the common bottlenose dolphin (CBD), determined by linear regression analysis (* indicates significance). The significant difference observed in Port Phillip Bay δ^13^C is likely confounded by sex of the specimen (with females being collected at earlier dates than males) rather than any temporal variation. No difference was observed in the δ^15^N of the Port Phillip Bay population and for either isotope value in the other SABD population in the Gippsland Lakes.

For the SABD populations, the Port Phillip Bay population displayed significantly higher δ^15^N (17.0‰ vs. 15.5‰) values than the Gippsland Lakes population (F_1, 12_ = 8.100, p = 0.015) (Figure 2). No significant difference was observed in the δ^13^C signature of the two SABD populations (F_1, 12_ = 2.961, p = 0.111).

There was an opposite trend in the value of δ^15^N in the two systems when comparing the potential prey items and the dolphin populations. The Gippsland Lakes dolphins had a lower δ^15^N than the Port Phillip Bay dolphins, whereas the Gippsland Lakes potential prey items had a higher δ^15^N than the Port Phillip Bay potential prey items. The Port Phillip Bay dolphins had an average δ^15^N 4.5‰ higher than the average Port Phillip Bay potential prey items δ^15^N. The Gippsland dolphins had an average δ^15^N only 1.3‰ higher than the average Gippsland Lakes potential prey items δ^15^N (Figure 2). The raw data is available in Data S1.

## Discussion

Despite the relatively cryptic nature of the dolphin species described in this study and the very small amounts of biological material available, we were able to determine possible differences in trophic relationships. This study represents the first information on the foraging ecology of the SABD. Also, it provides an additional line of evidence supporting genetic and morphological data that indicate the SABD is distinct from the CBD in Victoria, Australia. Differences in the stable isotope signatures were observed between the SABD and the CBD. This indicates that these two species are likely to forage in different areas and potentially consume different prey. Although sample sizes in this study are small and further investigation into these observed differences is required, this information adds greatly to our knowledge of this new species and provides an indication of the way forward in conserving this newly identified and potentially endangered, endemic species of dolphin.

Dietary segregation between the CBD and SABD is likely due to differences in habitat and habitat occupancy of the two species. In offshore waters, CBD occupy deep, cool waters and are rarely seen in coastal areas. Consequently, CBD is likely to feed on species which ultimately derive their energy from primary producers in the water column (euphotic phytoplankton). Conversely, the SABD is found predominantly in warm, shallow coastal waters and embayments, and in addition to euphotic sources, has access to prey items which may derive their energy from a variety of basal resources including bottom-living (benthic) primary producers such as macroalgae and seagrass. Furthermore, differences in habitat occupancy may explain the differences in isotopic signature between species. The SABD is believed to be resident to inshore areas of Victoria [13] whereas the CBD is most likely predominant in offshore areas. A study of bottlenose dolphins resident to Doubtful Sound in New Zealand found the population relied more heavily on benthic sources located inside of the Sound, rather than pelagic subsidise from outside of the Sound [41]. It is likely that the SABD is also strongly reliant on coastal benthic food webs found inside of Port Phillip Bay and the Gippsland Lakes, given the higher δ^13^C observed in the SABD compared to the CBD which displayed a lower, more pelagic δ^13^C signature. Additionally, differences in the diet of inshore and offshore populations of dolphin species have been described elsewhere in the world. Using the stable isotope ratios in teeth it was shown that offshore bottlenose dolphins in the Northwest Atlantic had a higher proportion of squid in their diet compared to inshore populations that fed predominately on fish [42]. These differences in both the foraging areas and lifestyles provide important supporting evidence for the stable isotope data in suggesting a likely difference in the energy base supporting dolphin productivity in the two species.

In addition, dietary differences between the two species may also be attributed to differences in size, with the CBD being considerably longer (∼3 m) than the SABD (∼2.5 m) (Charlton-Robb, *et al*., unpublished data). Larger animals are generally believed to be able to consume larger prey items due to the larger gape of their mouth [43], [44]. However, if this was the case and the larger CBD were feeding on a higher trophic level it would be expected that the CBD would exhibit a higher δ^15^N. Also, a difference in size of the predators is likely to affect their manoeuvrability and therefore their foraging capacity [45]. As marine vertebrates get larger they tend to favour less manoeuvrable prey and use different tactics to capture prey [45]. Further analyses using offshore prey items would allow comparisons between the offshore and inshore food webs. However, the distinction of the isotope signatures between the two species is strongly indicative that the two species do indeed forage in different areas and may feed on different prey. This data provides an additional line of evidence supporting the genetic and morphological data that the SABD is distinct from the CBD in Victorian waters, Australia.

As well as the interspecific differences observed in the δ^13^C and δ^15^N signature of the teeth, differences were also observed between populations of the SABD. The population in the Gippsland Lakes displayed a lower value for δ^15^N than the population in Port Phillip Bay. This implies that the Port Phillip Bay dolphins may feed on a higher trophic level than the Gippsland Lakes dolphins. This theory is also supported by comparisons of the dolphin isotopic signatures to that of potential prey items in each system. Typically, the amount of fractionation between trophic levels is approximately 3‰ per trophic level for δ^15^N [27], [28]. The Port Phillip Bay population was 4.5‰ higher for δ^15^N than the average signature of the potential prey items, whereas the Gippsland Lakes population was only 1.3‰ higher than the same potential prey species sampled in the Gippsland Lakes. Based on this we hypothesise that the Port Phillip Bay population may be feeding on these prey items as well as potentially some prey items that have a higher trophic level than the prey items sampled. Conversely, the Gippsland Lakes population may feed on the prey items sampled in addition to lower trophic level prey that will lower the average δ^15^N signature received.

Although the Gippsland Lakes and Port Phillip Bay are both shallow coastal environments, they do differ significantly in nature. Port Phillip Bay is a large drowned river system covering an area of approximately 1930 km^2^ [46]. The majority of the bay is 8 m deep with a relatively constant salinity and temperature. Conversely, the Gippsland Lakes is a 75 km long series of coastal lakes and lagoons that has large temperature fluctuations seasonally and with distance from the marine input [47]. The salinity of the Lakes also varies seasonally, being influenced by salt water entering from Bass Strait and variability in fresh water inputs from river inflows [47]. Both Port Phillip Bay and the Gippsland Lakes are connected to the offshore environment of Bass Strait located between Victoria and Tasmania, Australia (Figure 1). These environmental differences are likely to result in variation in basal resource availability and consequently, in the dominant fish species in the two waterways. It is likely that this variation in fish assemblages influences dolphin diet and hence stable isotope signatures. However, it cannot be ruled out that these physiochemical differences may also alter patterns of isotopic fractionation, and thus the dolphins are feeding on the same diet, and fractionation differences have resulted in the distinction of isotope signatures. Nitrogen isotope values are dependent on the source of nitrogen at the base of the food web and isotopic shifts are associated with nutrient transformation. For example, due to the large fractionation factor associated with nitrification, nitrate can have a very low isotope value if it is derived from nitrification of ammonium. If the food web is dependent on this nitrate, the low isotope value will be reflected in the food web. However, if denitrification predominates, a residual pool of ammonium will have a very high isotope value that is transferred to the food web. Thus, identical food webs can be offset by isotope shifts that occur during nitrogen transformations.

It is possible that the Gippsland Lakes food webs may be more dependent on nitrate with a lower nitrogen isotope value and the Port Phillip Bay food webs may be more reliant on ammonia with a higher nitrogen isotope value. If this is true, then it is possible differences in δ^15^N between the dolphin populations are as a result of differing basal nitrogen sources in the two systems, rather than differences in diet. However, this explanation appears unlikely. While prey items sampled in Gippsland Lakes had a higher average nitrogen signature than the same prey items sampled in Port Phillip Bay, the opposite trend was observed between the dolphins. This suggests that differences in basal nitrogen sources, if they exist, cannot account for the differences in stable isotope values for the dolphins. This suggests that the Port Phillip Bay dolphins may be feeding at a higher trophic level than the Gippsland Lakes dolphins.

It is important to note that interpreting these stable isotope results is made more challenging by the difficulties in establishing an appropriate baseline isotope value for dolphin prey in Port Phillip Bay and the Gippsland Lakes. A good baseline has to take into account changes in baseline isotope values over time scales relevant to the species of interest [48]. For long lived consumers such as marine mammals this is extremely difficult, especially when working with a tissue such as teeth where the isotopic signature represents the integrated signature over the life time of the animal (the average age of the animals in this study was 11 years- the oldest animal was 30 years). Ideally, we would be able to compare prey from the two locations which had a similar life span to dolphins, in order to account for any potential temporal shifts in basal δ^13^C and δ^15^N. There are no prey species which are as long-lived as dolphins, and as a result we have assumed that the δ^13^C and δ^15^N signatures of basal resource are broadly consistent through time.

In addition to the potential for baseline isotope values to differ through time, recent studies have also indicated the potential for baseline isotope values to differ spatially, even over relatively small scales in relation to factors such as salinity and temperature [49]. For marine mammals establishing an accurate baseline can be difficult in that they are highly mobile, and their distributions are not always well understood. We have taken a conservative approach here and assumed that the basal nitrogen sources do not differ. Never the less these constraints of our knowledge of baseline values mean that we cannot conclusively state based on our stable isotope results alone that the two populations differ in their trophic relationships.

Although there have been no prior dietary studies on these species, there is additional supporting evidence for the structure of the food webs proposed here. Stable isotope analysis of Little Penguins (*Eudyptula minor*) have found comparable values for the stable isotopic signatures of a number of the prey species considered here, and provides external validation of the values in this study [50]. Based on the penguin stable isotope signatures it appears that there is at least some degree of dietary overlap between the penguins and the dolphins in Port Phillip Bay. The magnitude and significance of that overlap is beyond the scope of this paper, but does identify the potential for competition between species of conservation concern. This competition has the potential to be intensified if environmental conditions act to reduce prey which are specific to each species. Human impacts of fishing could also act to increase competition between the two species, through removal of larger fish (which exceed penguin gape size, but can be fed on by dolphins). These potential interactions are speculative at this stage, but indicate the power of resolving food webs using stable isotope analysis to inform management.

**Figure 3:**
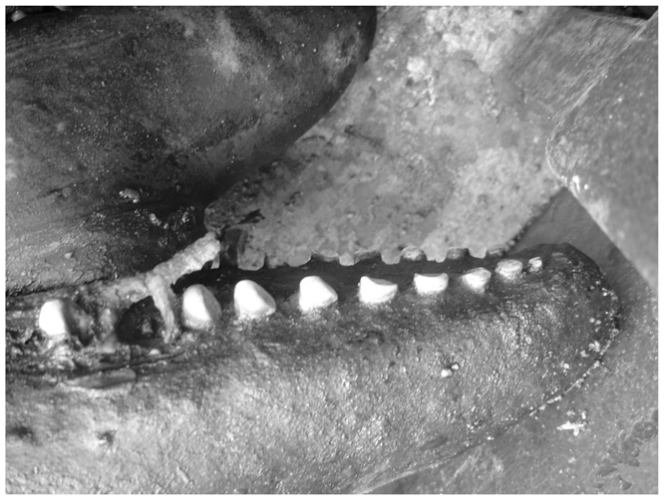
Tooth wear of the Southern Australian bottlenose dolphin in the Gippsland Lakes. It can be seen that the teeth are highly worn towards the front of the jaw.

**Figure 4:**
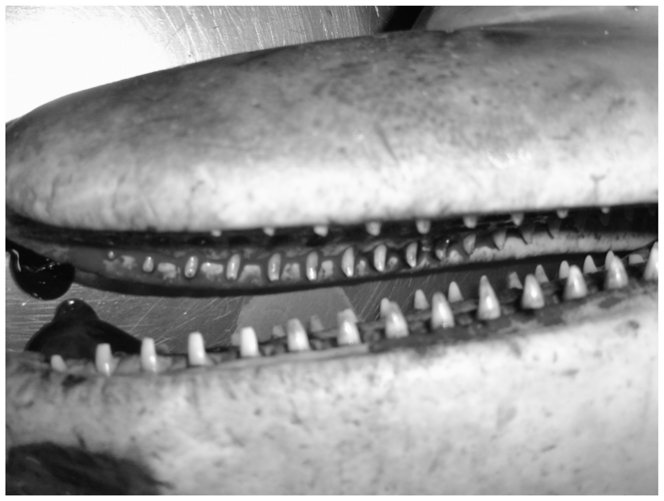
Tooth wear of the Southern Australian bottlenose dolphin in Port Phillip Bay. It can be seen that there is very low levels of tooth wear present.

In addition to isotopic differences, we have observed the active foraging strategies of the two populations to be distinctly different (Dolphin Research Institute; Charlton-Robb, *et al.*, unpublished data). In Port Phillip Bay, the dolphins are commonly observed herding schools of fish into bait balls at the surface. In the Gippsland Lakes feeding behaviour is based to a great extent on cooperatively working to herd fish into the shallows. The idea that the two populations are consuming different prey items is also supported by observations of different patterns of tooth wear in the two populations (Dolphin Research Institute; Charlton-Robb, *et al.*, unpublished data). A large amount of tooth wear was observed at the front and back of the jaw of the Gippsland Lakes dolphins (Figure 3), whereas the teeth in the centre of the jaw have a very little amount of wear. This pattern of tooth wear is distinctly different from that of the Port Phillip Bay dolphins which display very low levels of tooth wear throughout the whole jaw (Figure 4). It is possible that the front teeth of the Gippsland Lakes animals become worn when removing prey from the sediment. Taken in combination with the stable isotope data, the nature of food likely to be available in the two habitats, differences in tooth wear and observed differences in feeding behaviour, we believe that there is evidence for differences in diet between the two populations. However future studies should include sampling of a wider range of basal resources and work with a tissue such as skin or blubber that has a higher turnover rate to clearly determine if this is the case.

### Conclusion

This study provides the first information on the foraging ecology of the SABD. Using stable isotope analysis of dolphin teeth and prey items, clear differences in the foraging ecology of the SABD and CBD are apparent, as well as possible differences in trophic level of foraging between the two small populations of the SABD present in Victoria, Australia. The differences in stable isotope signatures between the SABD and the CBD provide an additional line of evidence that the SABD is distinct from CBD in Victoria, Australia. The likely dietary difference between populations of SABD is important as it may require the two populations of this recently identified species to be managed separately. This study clearly illustrates the potential for the use of stable isotope analysis to resolve trophic relationships of species, even when they are cryptic, and occupy habitats where direct observation of feeding is not possible. There is considerable potential to apply this to a number of species of conservation interest, including in environments where there is concern that changes in habitat may be altering niches and disrupting species barriers. In broader ecological terms, there is also the potential to map dietary differences determined by stable isotopes on to measures of availability of prey in order to understand the potential for dietary specialisation to maintain species barriers, or to act to make hybrids unfit, through a failure to effectively occupy a single trophic niche within a habitat. Finally, our use of teeth in this study illustrates the potential of stable isotope analysis to trace changes in diets through time, attribute trophic relationships to extinct species where sub-fossil or museum material is available, and identify key prey-predator relationships which may need to be targeted to restore ecosystems.

## Materials and Methods

### Sample collection

Samples were collected from both the National Museum Victoria (n = 28) and recently stranded individuals (n = 5) across Victoria, that had been identified as ‘*Tursiops’* species, as the SABD had historically been identified and documented as both *Tursiops truncatus* and *T. aduncus* (Table 4 and Table 5). Genetic analysis as well as skull morphology analysis was used to confirm the species of the specimens (Charlton-Robb *et al*., unpublished data). All museum specimens used were from beach-cast animals collected on Victorian beaches over the last 40 years. One tooth from 28 dolphins was collected from the National Museum Victoria (specimens dating between 1967 and 2006). Only non preserved specimens were collected as preservatives such as ethanol could influence the isotopic signature. One tooth was collected from five dead beach cast animals (from between 2007 and 2008), during the de-flensing process. Teeth with the least wear were chose to allow for complete analyses across the dolphins life. External gum material was removed from the outside of the teeth using a brush or forceps. Samples were stored in a dry environment at room temperature. A single tooth was selected from each animal for analysis as bottlenose dolphins have been shown to display a low variation in stable isotopes signatures within an animal [20].

**Table 4:** Specimen information for the southern Australian bottlenose dolphins.

Museum Code	Location	Year	Sex	Age	Population	Stomach contents	δ^13^C	δ^15^N	C:N
Southern Australian bottlenose dolphin									
**Gippsland Lakes**									
C35986	Mitchell River	2006	Female	20	Gippsland Lakes	Empty stomach	−14.3	15.2	3:3
C29582	Tom's creek	1986	Female	20	Gippsland Lakes	Unknown	−13.2	15.2	3:3
C35987	Holland's landing	2006	Male	11	Gippsland Lakes	Empty stomach	−14.5	15.6	3:3
C35965	Lake Wellington	2006	Male	9	Gippsland Lakes	Empty stomach	−15.8	16.0	3:3
C35985	Blonde Bay	2006	Male	8	Gippsland Lakes	Empty stomach	−15.4	15.2	3:3
C35966	Lake Wellington	2006	Male	30	Gippsland Lakes	Empty stomach	−15.3	15.8	3:3
C29463	Secombe	1984	Unknown	25	Gippsland Lakes	Unknown	−15.2	14.6	3:7
C35968	Poddy Bay	2006	Unknown	11	Gippsland Lakes	Empty stomach	−14.9	14.3	3:4
**Port Phillip Bay**									
C24944	Elwood	1967	Female	Unknown	Port Phillip Bay	Squid beaks	−13.4	15.5	3:4
C29577	Safety Beach	1985	Female	10	Port Phillip Bay	Unknown	−13.8	14.6	3:3
C29461	Port Melbourne	1984	Female	11	Port Phillip Bay	Unknown	−13.8	18.5	3:3
Beaumaris	Beaumaris	2008	Male	21	Port Phillip Bay	Empty stomach	−15.1	18.6	3:2
Point Henry	Point Henry	2008	Male	13	Port Phillip Bay	Empty stomach	−15.0	17.8	3:3
C28760	Sandringham	1992	Unknown	13	Port Phillip Bay	Unknown	−13.4	17.0	3:3
**Population unknown**									
C29586	Rippleside	1991	Male	12	Unknown	Unknown	−13.9	16.6	3:3
C29587	Kennedy's point	1992	Male	9	Unknown	Unknown	−12.7	14.3	3:4

Museum code represents the Melbourne museum number for each specimen or the collection location for the specimen. Location and Year indicate the point on the Victorian coastline and year of collection. Age was determined by thin sectioning and counting dentinal layers of teeth. Where stomach contents are Unknown indicates that no data was collected at the year of specimen collection. C: N is represented as a mass ratio.

**Table 5:** Specimen information for common bottlenose dolphins.

Museum Code	Location	Year	Sex	Age	Population	Stomach contents	δ^13^C	δ^15^N	C:N
Common bottlenose dolphin									
C29584	Torquay	1988	Female	2	-	Unknown	−15.9	15.2	3:3
C31643	Unknown	Unknown	Female	12	-	Unknown	−15.3	14.8	3:3
C29460	Sutton Rocks	1984	Female	11	-	Unknown	−15.3	12.8	3:3
C23490	Lorne	1979	Female	Unknown	-	Unknown	−15.1	15.4	3:3
Point Ricardo	Point Ricardo	2007	Female	11	-	Squid beaks	−15.4	16.2	3:1
Kilarney Beach	Kilarney Beach	2008	Female	9	-	Fish vertebrae	−15.5	15.0	3:2
C29581	Port Fairy	1986	Male	4	-	Unknown	−15.5	14.2	3:3
C24990	Kilarney Beach	1981	Male	2	-	Unknown	−15.2	15.9	3:4
C29585	Wild Dog creek	1990	Male	9	-	Unknown	−15.2	13.9	3:3
C29580	Murrells beach	1986	Male	14	-	Unknown	−15.2	15.6	3:3
C35969	Phillip Island	2006	Male	8	-	Empty stomach	−15.5	14.4	3:3
C35984	Port Fairy	2006	Male	8	-	Empty stomach	−15.6	15.8	3:3
Cape Conran	Cape Conran	2008	Unknown	Unknown	-	Prawn and squid	−13.4	16.1	3:2
C35947	Cape Conran	2004	Unknown	8	-	Unknown	−17.1	14.8	3:5
C7799	Lorne	1967	Unknown	3	-	Unknown	−15.5	14.6	3:3
C24987	Lorne	1967	Unknown	4	-	Unknown	−15.9	16.0	3:5
C28677	Wilson's Prom.	1991	Unknown	1	-	Unknown	−15.9	14.5	3:3

Museum code represents the Melbourne museum number for each specimen or the collection location for the specimen. Location and Year indicate the point on the Victorian coastline and year of collection. Age was determined by thin sectioning and counting dentinal layers of teeth. Where stomach contents are Unknown indicates that no data was collected at the year of specimen collection. C: N is represented as a mass ratio.

As age of the specimen and year of collection could be potential confounding variables in the analysis of foraging ecology, the age of each individual was determined. Teeth were wafered, decalcified, thin sectioned and stained, before being mounted onto a slide in order to facilitate counting of the growth layer groups [51]. Two people completed triple blind counts of each of the specimens and then the average of these counts was used as the age of the animal.

In order to compare the trophic level of foraging between the two populations of SABD, the same fish species from both systems were collected from local fishermen. Six of the dominant fish species in both systems (Snapper (*Pagrus auratus*), Yellowfin Bream (*Acanthopagrus australis*), Squid (*Nototodarus gouldi*), Silver Trevally (*Pseudocaranx dentex*), Flathead (*Platycephalus* sp.) and, Garfish (*Hyporhampus* sp.)) were chosen (Table 1). All samples were stored at −20°C prior to analysis.

### Stable Isotope Analysis

For dietary items, approximately 1 cm^3^ section of muscle tissue was cut from the dorsal surface of each fish. For squid, a 1 cm^3^ section of the hood was sampled. The skin and scales were removed from the sample. Samples were then frozen at −80°C until being freeze dried for 4.5 days. The dried samples were ground into a fine powder using a mortar and pestle, and 1 mg of the powder was capsulated in tin for analysis.

For the dolphin samples, teeth were cut in half using a wet blade diamond saw. Once sectioned, a scalpel was used to remove any internal pulp material that may contaminate analysis. The section of tooth was crushed under 10t/cm pressure. The crushed tooth was then soaked in 32% HCl for approximately 24 hours to remove biogenic carbonates [39]. Many studies rinse the sample with distilled water after acidification to remove any acid remains. As this can result in the loss of some organic carbon components in the sample [52], [53], samples were not rinsed after acidification and instead were placed in an oven at 60°C for a week to allow excess HCl to evaporate. The samples were then placed into the −80°C freezer overnight before being freeze dried for three days to dehydrate the sample and facilitate grinding into a fine powder using a mortar and pestle. Approximately 3 mg of the tooth powder was then weighed into a tin capsule for analysis.

Variability in the lipid content of samples has the potential to bias stable isotope results. This is because lipids are depleted in ^13^C relative to other organic molecules [54]. It has been shown that the bias introduced by lipids increases as the concentration of lipids in the sample increases. It is therefore not necessary to account for lipids if the lipid content in the sample is low (below around 5% lipid or a C:N ratio of <3.5 for aquatic animals) [54]. As direct chemical removal of lipids can affect the δ^15^N of the tissue [55], no chemical treatment of the samples was applied. Additionally, after receiving the C:N ratios of the samples (Table 1, 4 and 4) it was determined that the lipid content of the samples was low so no further normalisation of the data to account for the lipid affect was required.

All samples in tin capsules were sent to the Stable Isotope Analysis Laboratory at Griffith University in Queensland for analysis. Samples were oxidised at high temperatures, then combusted in a EuroEA 3000 elemental analyser. The resulting N_2_ and CO_2_ gases were separated chromatographically and fed into an IsoPrime isotope ratio mass spectrometer. The isotopic standards used were ANU sucrose for carbon and atmospheric air for nitrogen. Isotope values are expressed as:
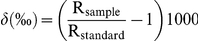



### Data Analysis

The species of the specimens was determined based on both genetics and skull morphology (Charlton-Robb *et al*., unpublished data). Whether the specimens of the SABD were from the Gippsland Lakes or the Port Phillip Bay population was determined based on genetic haplotypes that are known to be restricted to each region based on live animal biopsy sampling (Charlton-Robb *et al.*, unpublished data). Means for prey data were determined using Microsoft Excel 2007. Differences between species (SABD and CBD) and SABD populations (Port Phillip Bay and Gippsland Lakes) were analysed using factorial ANOVA with Helmert contrasts and Type III sum of squares to control for the unequal sample sizes present in this study. Relationships between the year of collection and the age of the animal were determined using Linear Regression. Analyses were conducted using the statistical software R 2.6.1 [56] with an alpha value of 0.05.

## Supporting Information

Data S1 The raw data is presented in two tabs, one showing the prey data, the other showing the dolphin data associated with this study.
(XLS)

## References

[1] Schluter D (2009). Evidence for ecological speciation and its alternative.. Science.

[2] Abbott I, Abbott LK, Grant PR (1977). Comparative Ecology of Galapagos ground finches (*Geospiza gould*): Evaluation of the importance of floristic diversity and interspecific competition.. Ecol Monogr.

[3] Jensen KR (1997). Evolution of the Sacoglossa (Mollusca; Opisthobranchia) and the ecological associations with their food plants.. Evol Ecol.

[4] Schluter D, McPhail JD (1992). Ecological character displacement and speciation in stickleback.. Am Nat.

[5] Ford JKB, Ellis GM, Barrett-Lennard LG, Morton AB, Palm RS (1998). Dietary specialization in two sympatric populations of killer whales (*Orcinus orca*) in coastal British Columbia and adjacent waters.. Can J Zoolog.

[6] Hoelzel AR, Dover GA (1991). Genetic differentiation between sympatric killer whale populations.. Heredity.

[7] Morin PA, Archer FI, Foote AD, Vilstrup J, Allen EE (2010). Complete mitochondrial genome phylogeographic analysis of killer whales (*Orcinus orca*) indicates multiple species.. Genome Res.

[8] Barros NB, Wells RS (1998). Prey and feeding patterns of resident bottlenose dolphins (*Tursiops truncatus*) in Sarasota Bay.. Florida J Mammal.

[9] Blanco C, Salomon O, Raga JA (2001). Diet of the bottlenose dolphin (*Tursiops truncatus*) in the western Mediterranean Sea.. J Mar Biol Assoc UK.

[10] Gannon DP, Waples DM (2004). Diets of coastal bottlenose dolphins from the U.S. mid-Atlantic coast differ by habitat.. Mar Mammal Sci.

[11] Santos MB, Fernandez R, Lopez A, Martinez JA, Pierce GJ (2007). Variability in the diet of bottlenose dolphin, *Tursiops truncatus* in Galician waters, north-western Spain, 1990-2005.. J Mar Biol Assoc UK.

[12] Moller LM, Beheregaray LB (2006). Coastal bottlenose dolphins from south-eastern Australia are Tursiops aduncus according to sequences of the mitochondrial DNA control region.. Mar Mammal Sci.

[13] Hale P (2002). Interactions between dolphins and vessels in Port Phillip Bay..

[14] Charlton K, Taylor AC, McKechnie SW (2006). A note on divergent mtDNA lineages of bottlenose dolphins from coastal waters of southern Australia.. J Cetacean Res Man.

[15] Moller LM, Bilgmann K, Charlton-Robb K, Beheregaray L (2008). Multi-gene evidence for a new bottlenose dolphin species in southern Australia.. Mol Phylogenet Evol.

[16] Kingston SE, Adams LD, Rosel PE (2009). Testing mitochondrial sequence and anonymous nuclear markers for phylogeny reconstruction in a rapidly radiating group: molecular systematics of the Delphininae (Ceatcea: Odontoceti: Delphinidae).. http://dx.doi.org/10.1186/1471-2148-9-245.

[17] Real J (1996). Biases in diet study methods in the Bonelli's Eagle.. J Wildl Manage.

[18] Dickman CR, Huang C (1988). The reliability of faecal analysis as a method for determining the diet of insectivorous mammals.. J Mammal.

[19] Williams MJ (1981). Methods for analysis of natural diet in portunid crabs (Crustacea: Decapoda: Portunidae).. J Exp Mar Biol Ecol.

[20] Walker JL, Macko S (1999). Dietary studies of marine mammals using stable carbon and nitrogen isotopic ratios of teeth.. Mar Mammal Sci.

[21] Meynier L, Pusineri C, Spitz J, Santos MB, Pierce GJ (2008). Intraspecific dietary variation in the short-beaked common dolphin (*Delphinus delphis*) in the Bay of Biscay: Importance of fat fish.. Mar Ecol Prog Ser.

[22] Tollit DJ, Steward MJ, Thompson PM, Pierce GJ, Santos MB (1997). Species and size differences in the digestion of otoliths and beaks: Implications for estimates of pinniped diet composition.. Can J Fish Aquat Sci.

[23] Lesage V, Hammill MO, Kovacs KM (2001). Marine Mammals and the community structure of the estuary and Gulf of St. Lawrence, Canada: evidence from stable isotope analysis.. Mar Ecol Prog Ser.

[24] Yonezaki S, Kiyota M, Takemura A (2003). Size distribution of the hard remains of prey in the digestive tract of northern fur seal (*Callorhinus ursinus*) and related biases in diet estimation by scat analysis.. Mammal Stud.

[25] Grellier K, Hammond PS (2006). Robust digestion and passage rate estimates for hard parts of grey seals (*Halichoerus grypus*) prey.. Can J Fish Aquat Sci.

[26] Nino-Torres CA, Gallo-Reynoso JP, Galvan-Magana F, Escobar-Briones E, Macko SA (2006). Isotopic analysis of δ^13^C, δ^15^N and δ^34^S. “A feeding tale” in the teeth of the long beaked common dolphin, *Delphinus carpensis*.. Mar Mammal Sci.

[27] DeNiro MJ, Epstein S (1981). Influence of diet on the distribution of nitrogen isotopes in the animal.. Geochim Cosmochim Ac.

[28] Hobson KA, Schell DM, Renouf D, Noseworthy E (1996). Stable carbon and nitrogen fractionation between diets and tissues of captive seals: Implications for dietary reconstruction involving marine mammals.. Can J Fish Aquat Sci.

[29] DeNiro MJ, Epstein S (1978). Influences of the diet on the distribution of carbon isotopes in the animal.. Geochim Cosmochim Ac.

[30] Clementz MT, Koch PL (2001). Differentiating aquatic mammal habitat and foraging ecology with stable isotopes in tooth enamel.. Oecologia.

[31] Aurioles D, Koch PL, Le Boeuf BJ (2006). Differences in foraging location of Mexican and Californian elephant seals: Evidence from stable isotopes in pups.. Mar Mammal Sci.

[32] France RL (1995). Carbon-13 enrichment in benthic compared to planktonic algae: food web implications.. Mar Ecol Prog Ser.

[33] Hobson KA, Sease JL, Merrick RE, Piatt JF (1997). Investigating trophic relationships of Pinnipeds in Alaska and Washington using stable isotope ratios of nitrogen and carbon.. Mar Mammal Sci.

[34] Kelly JF (2000). Stable isotopes of carbon and nitrogen in the study of avian and mammalian trophic ecology.. J Zool.

[35] Kurle CM, Gudmundson CJ (2007). Regional differences in foraging of young-of-the-year Steller sea lions *Eumetopias jubatus* in Alaska: stable carbon and nitrogen ratios in blood.. Mar Ecol Prog Ser.

[36] Cerling TE, Harris JM, Passey BH (2003). Diets of east African Bovidae based on stable isotope analysis.. J Mammal.

[37] McIllwee AP, Johnson CN (1998). The contribution of fungus to the diets of three mycophagous marsupials in Eucalyptus forests, revealed by stable isotope analysis.. Func Ecol.

[38] Hilderbrand GV, Farley SD, Robbins CT, Hanley TA, Titus K (1996). The use of stable isotopes to determine the diets of living and extinct bears.. Can J Zoo.

[39] Knoff A, Hohn A, Macko S (2008). Ontogenetic diet changes in bottlenose dolphins (*Tursiops truncatus*) reflected through stable isotopes.. Mar Mammal Sci.

[40] Hobson KA, Sinclair EH, York AE, Thomason JR, Merrick RE (2004). Retrospective isotopic analysis of Steller sea lion tooth annuli and sea bird feathers: A cross taxa approach to investigating regime and dietary shifts in the Gulf of Alaska.. Mar Mammal Sci.

[41] Lusseau SM, Wing SR (2006). Importance of local production versus pelagic subsidies in the diet of an isolated population of bottlenose dolphins *Tursiops* sp.. Mar Ecol Prog Ser.

[42] Walker JL, Potter CW, Macko SA (1999). The diets of modern and historic bottlenose dolphin populations reflected through stable isotopes.. Mar Mammal Sci.

[43] Menard F, Labrune C, Shin Y, Asine A, Bard F (2006). Opportunistic predation in tuna: a size based approach.. Mar Ecol Prog Ser.

[44] Persson L, Andersson J, Wahlstrom E, Eklov P (1996). Size specific interactions in lake systems: predator gape limitations and prey growth rate and mortality.. Ecol.

[45] Webb PW, Buffrenil VD (1990). Locomotion in the biology of large aquatic vertebrates.. T Am Fish Soc.

[46] Hewitt CL, Campbell ML, Thresher RE, Martin RB, Boyd S (2004). Introduced and cryptogenic species in Port Phillip Bay, Victoria, Australia.. Mar Biol.

[47] Poore GCB (1982). Benthic Communities of the Gippsland Lakes, Victoria.. Mar Freshwater Res.

[48] Post DM (2002). Using stable isotopes to estimate trophic position: Models, methods, and assumptions.. Ecol.

[49] Jennings S, Warr KJ (2003). Environmental correlates of large scale temporal variation in the δ^15^N of marine animals.. Mar Bio.

[50] Chiaradia A, Forero MG, Hobson KA, Cullen JM (2010). Changes in diet and trophic position of a top predator 10 years after a mass mortality of a key prey.. ICES J Mar Sci.

[51] Evans K, McKenzie J, McIntosh R, Kemper C (2007). Workshop on age determination of marine mammals using tooth structure..

[52] Jacob U, Mintenbeck K, Brey T, Knust R, Beyer K (2005). Stable isotope food web studies: for standardized sample treatment.. Mar Ecol Prog Ser.

[53] Carabel S, Godinez-Dominguez E, Verisimo P, Fernandez L, Freire J (2006). An assessment of sample processing methods for stable isotope analyses of marine food webs.. J Exp Mar Biol Ecol.

[54] Post DM, Layman CA, Arrington DA, Takimoto G, Quattrochi J (2007). Getting to the fat of the matter: models, methods and assumptions for dealing with lipids in stable isotope analyses.. Oecologia.

[55] Pinnegar JK, Polunin NVC (1999). Differential fractionation of delta c-13 and delta n-15 among fish tissue: implications for the study of trophic interactions.. Funct Ecol.

[56] R Development Core Team (2008). R: A language and environment for statistical computing.. http://www.R-project.org.

